# IWOA-LSTM based intrinsic structural identification of steel fiber concrete

**DOI:** 10.1038/s41598-025-08867-6

**Published:** 2025-07-17

**Authors:** Ping Li, Jie Feng, Shiwei Duan

**Affiliations:** 1 School of Management Science and Engineering, Anhui University of Technology, Maanshan, 243032 China; 2 School of Mechanical Engineering, Anhui University of Technology, Maanshan, 243032 China

**Keywords:** Whale optimisation algorithm, LSTM neural network, Steel fiber concrete, High temperature, Damage, Civil engineering, Composites

## Abstract

Fracture damage in steel fiber concrete (SFRC) is a developmental process in which deformation and damage are coupled with each other. In order to accurately identify the high-temperature constitutive model taking into account the damage evolution, a high-temperature constitutive identification model using the Improved Whale Algorithm (IWOA) optimised Long Short-Term Memory (LSTM) neural network is presented. Firstly, the Laplace crossover operator strategy, the optimal neighbourhood perturbation strategy, the adaptive weighting strategy and the updating strategy of the variables helix position are introduced to solve the problems of the Whale Optimisation Algorithm (WOA) in relation to its slow convergence rate and its tendency to fall into the locally optimal solution. The supremacy of the IWOA has been demonstrated by comparing IWOA with WOA, Crown Porcupine Optimisation Algorithm (CPO), Butterfly Optimisation Algorithm (BOA) and Grey Wolf Optimisation Algorithm (GWO) in terms of optimisation search. Secondly, based on the experimental data, LSTM model, WOA-LSTM model and IWOA-LSTM model were established, where the *MSE* of IWOA-LSTM model was improved by 47.66% and 65.60% compared to WOA-LSTM model as well as LSTM model. Finally, the constitutive identification model of SFRC using the IWOA-LSTM model was applied to decouple the damage and plastic strain by the comparative analysis of the measured curves and the prediction curves without the damage, so that the damage and its evolution law of steel fiber concrete at different temperatures (*T* = 200 °C, *T* = 400 °C and *T* = 520 °C) were obtained. The degree of approximation between the IWOA-LSTM model’s prediction and experimental data shows that the trained model has a high learning accuracy and good generalization capability, making it appropriate for use in structural engineering applications.

**Supplementary Information:**

The online version contains supplementary material available at 10.1038/s41598-025-08867-6.

## Introduction

Concrete is one of the most widely used construction materials and plays an important role in infrastructure development^1,2^. Randomly and evenly dispersed steel fibers may effectively hinder the development of micro fractures and prevent the creation of macro cracks, consequently increasing the material’s stability and toughness^3^. Concrete structures are not only subjected to a variety of normal loads throughout their lifetime, but are also exposed to accidental fires caused by a variety of causes, such as fuel and vapour explosions. Building fire accidents occur from time to time and fire has always been one of the serious disaster threats to building structures^4,5^. Concrete that has experienced fire or high temperatures undergoes a series of physical and chemical changes internally, leading to a deterioration of its mechanical properties, which can threaten the life of the building and the safety of people. Therefore, it is of great importance to establish a constitutive identification model for high temperature damage of steel fiber concrete in place to accurately predict the thermodynamic response of steel fiber concrete.

More and more scholars have studied the damage constitutive models of concrete, the conventional constitutive model may be loosely split into two categories, one of which is the constuctive model based on the theory of composite reinforcement and microscopic damage mechanics. For example, Hameed et al.^6^ suggested a damage constitutive model based on the damage behaviour law of plain and fiber reinforced concrete for the prediction of SFRC, considering the fiber-matrix bond damage of sliding fibers. Wang et al.^7^ used the 4D CT in-situ experimental technique to study the fine structural variations and mechanical behavior of steel fiber concrete during cyclic uniaxial compression loading, and they proposed a revised stiffness degradation damage constitutive model that takes into account steel fiber distribution orientation and ITZ characteristics. Yu et al.^8^ suggested a model for the fracture behaviour of SFRC by using a discrete-continuous coupled finite element method to accurately follow the microscopic cracks during the bending process, taking into account key factors such as the spatial positioning of coarse aggregate and steel fibers, fiber content, length and diameter, and bonding properties at the interfacial transition zone (ITZ) of the fiber mortar. The second kind is an image-only constitutive model which is based on the macromechanical response of SFRC materials and the experimental data fitting and correlation analysis. For example, Bi et al.^9^ studied the influence of the volume percentage of steel fibers as well as the matrix strength on the mechanical characteristics of concrete materials, improved the HJC model and proposed a constitutive model suitable for SFRC materials. Chen et al.^10^ conducted compression and splitting tensile tests on SFRC with various admixtures, discussed the strain hardening effectiveness and the relationship between the energy dissipation of the specimens, and proposed a constitutive model for SFRC taking into account the damage evolution. In order to provide a form of damage evolution equation that is applicable to concrete materials under high temperature settings, Wen et al.^11^ conducted compression experiments of concrete materials at different temperatures (20℃, 200℃ and 400℃).

The results of the above research on the traditional constitutive model provide a valuable reference for the designing and application for steel fiber concrete constructions, and the established constitutive model is a mathematical equation expressed as an explicit function. However, there are several impacts on the characteristics of steel fiber concrete materials, which are often coupled (e.g. temperature, strain and damage), making it difficult to determine the material parameters accurately, and even impossible to determine some of the parameters (e.g. damage) directly. In addition, when analysing the data, different mathematical forms can be obtained by using different formulas for the regression fit (exponential fit, polynomial fit, etc.). If too many parameters are included, the solution of the mathematical equations is too complex and lacks practicality. If fewer parameters are included, the accuracy of the model is greatly reduced, it cannot fully reproduce the experimental data and repeatability is poor. Therefore, there is currently no easy application in engineering that can be used to accurately and comprehensively characterise the factors influencing the concrete material performance and the coupled effect of the factors in the constitutive model.

As computers have developed, machine learning methods have been gradually introduced into the field of civil engineering materials research by many scientists because of their high information processing capabilities, but most of the related research has focused on the prediction of concrete strength^12–15^ and image damage identification^16–18^. Ta, Q. A parametric study of image-based crack identification for orthotropic anisotropic steel bridge decks using captured images with complex backgrounds. The results show that the trained ACDN model can identify fatigue cracks and the accuracy of the crack detection results is improved by optimising the training parameters^16^. Ghaboussi et al.^19^ proposed the first use by neural network theory to construct constitutive models of materials. Afterwards this type of model has been continuously applied by many scholars to the constitutive model of soil, concrete and other materials^20–22^. For example, Wang et al.^23^ identified the intrinsic response and damage evolution laws of polymers through various input-output modes based on SHPB test data. Xu et al.^24^ investigated polymer constitutive models with and without damage evolution using a new approach that combines experimental techniques with a back-propagation (BP) neural network procedure. Ning et al.^25,26^ proposed an Artificial Neural Network (ANN) model based on the Back Propagation (BP) method for predicting concrete damage behavior. By comparing prediction accuracy, results show that the ANN model performs better than the commonly utilized experience equations. Zaidi et al.^27^ applied the Levenberg-Marquardt (LM) algorithm for constructing an ANN to predict the residual stress-strain curves of plain and fiber concrete under axial compression after fire. The findings show good agreement of the predicted stress-strain curves with the actual experimental stress-strain curves. Neural networks have advantages over traditional analysis methods in meeting the accuracy, complexity, and stability of material constitutive relationships. Based on neural network methodology to determine the material constitutive model, not dependent on the failure mechanism, which has not yet been well defined, no need for complex parameter analysis, through the given sample data to learn, directly extract the rules from them to get the required data, to ensure the accuracy while saving a lot of time. Currently, the concrete constitutive identification model is applied more BP neural network, but BP as the earliest known and most widely used a kind of neural network, its own also exists certain limitations and shortcomings, low identification accuracy, poor generalisation ability and so on. Recurrent Neural Networks (RNN) are able to maintain the previous state of information when processing sequential data, and have the function of ‘memory’, which has demonstrated great performance in forecasting the performance of construction materials^28^. LSTM, as a variant of RNN, is time sensitive and by introducing a gating mechanism, it overcomes the limitation of RNN in solving long term dependency problems and can increase the accuracy of the model’s prediction. Recently, neural network research has been carried out in many fields, such as earthquake risk prediction, image processing, medicine, finance and so on, and has achieved certain results. As such, Xu et al.^29^ suggested a LSTM neural network for predicting the seismic response of nonlinear structures of arbitrary length and sampling rate. The findings show that the suggested LSTM model appropriately reproduces the global and specific characteristics of the time histories of four kinds of structural response datasets, with good accuracy and generalisability. But LSTM has numerous parameters like neuron count, learning rate, thresholds and so on. These hyperparameters have a big influence on the results of its prediction and it will take a lot of time to take the values only by experience. Zhang et al.^30^ used the WOA algorithm to optimise the number of hidden neurons, time step and batch size of the LSTM to construct the WOA-LSTM model that predicted the amount of gas inflow. The results indicate that when the WOA-LSTM model is contrasted with the LSTM, RNN and BP neural network models, the WOA-LSTM is the best among the above models.

In summary, there are few studies that incorporate machine learning on damage constitutive models for steel fiber concrete materials under high temperature conditions, mostly focusing on single models such as BP neural networks. From the point of view of concrete damage accumulation, concrete is a kind of memory material, with the ‘memory’ that the previous load has an effect on the subsequent loads, and the stress-strain relationship of concrete presents nonlinear characteristics, while the traditional neural network is less efficient in dealing with time series data. LSTM can effectively predict data with nonlinear and temporal characteristics^31, 32^. However, LSTM has limitations in parameter selection, and the choice of parameter values has a substantial effect on prediction performance, and choosing an intelligent algorithm to optimise the parameters can increase the accuracy of the prediction^33^. When looking for the best neural network parameter solutions, traditional meta-heuristic optimisation techniques are prone to falling into local optimisation mode.

To deal with these challenges, we try to make some improvements to the current study. As a result, this research offers an Improved Whale Algorithm (IWOA)-optimized Long Short-Term Memory (LSTM) neural network as a constitutive identification model for steel fiber concrete at high temperatures. According to the principle of the Whale algorithm, the Laplace cross operator strategy, the optimal neighbourhood perturbation strategy, the adaptive weighting strategy and the variable helix position updating strategy are used to overcome the original shortcomings. To verify the optimality finding ability of IWOA, WOA, CPO, BOA and GWO algorithms, fifteen benchmark testing functions in CEC2005 dataset are selected and compared with them, IWOA algorithm has high accuracy and fast convergence speed. LSTM model, WOA-LSTM model and IWOA-LSTM model were established, and it can be seen from the error analysis that the prediction outcome of the IWOA-LSTM model is more accurate, and the prediction error can be guaranteed to be less than 0.4 in both cases of considering damage and not considering damage. The damage evolution curves of SFRC at various temperatures were obtained using the IWOA-LSTM model for the steel fiber concrete constitutive identification model, decoupling of damage and plastic strain, which confirmed the validity and excellence of the IWOA-LSTM high-temperature constitutive identification model for steel fiber concrete established in this paper. In addition, this paper only considered the high-temperature constitutive identification of concrete under quasi-static conditions, and the high-temperature constitutive identification at impact loading is not within the scope of this study.

## Materials and test

**Fig. 1:**
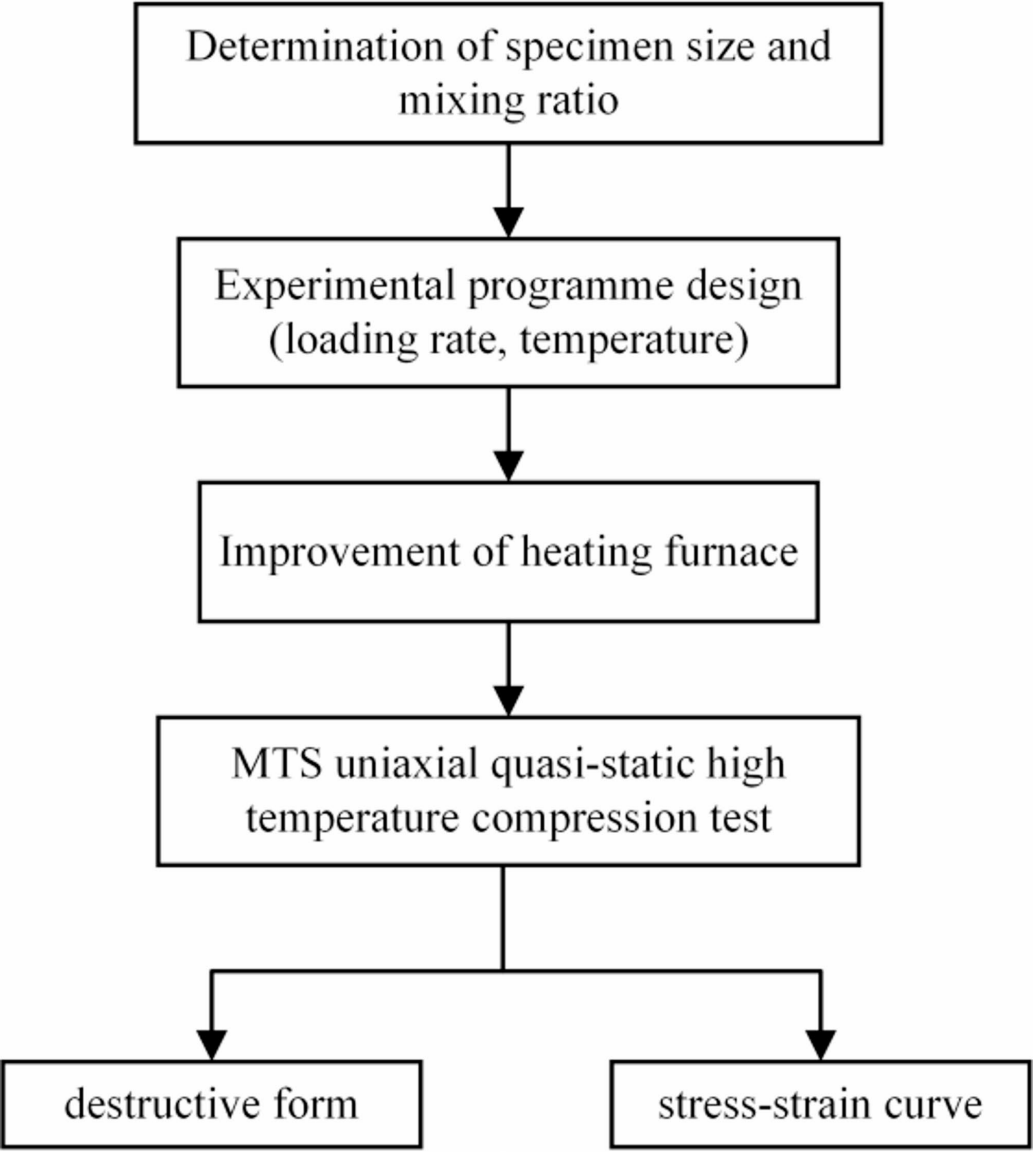
Flow chart of experimental design.

Raw material preparation for the test: The specimen is a cylinder with the size of φ50mm×100 mm. Cement selection of compressive strength of 42.5 MPa slag cement; coarse aggregate selected for 5–10 mm crushed stone; fine aggregate selected for the fineness modulus of 2.3 sand; steel fiber is used in the length of 6 mm, the diameter of 0.175 mm copper-plated surface of the ultra-fine, ultra-short steel fiber. The static load uniaxial compression tests in this paper were performed on an electro-hydraulic servo compression tester, model MTS810, with a high temperature heater installed to suit the specimens heating requirements in this paper. The design flow of the experiment is shown in Fig. 1. A complete description of how to prepare and maintain the specimens, how to heat the specimens, as well as the experimental set-up and experimental procedure can be seen in Li et al.^34^.

**Fig. 2:**
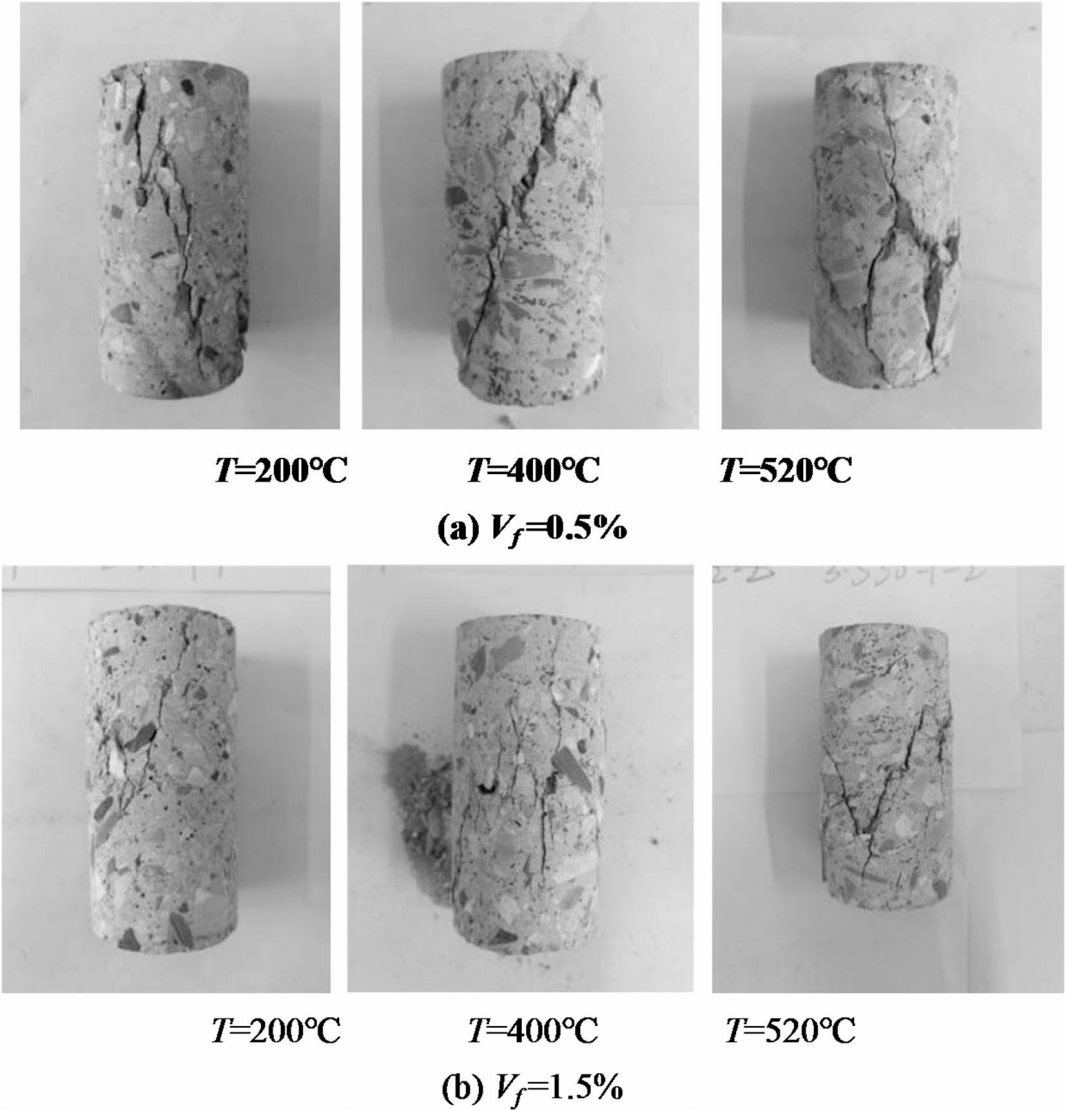
Damage morphology of SFRC at different temperatures.

Sample heating process, with the increase in temperature, the sample color will change, the temperature reaches 200 ℃ before the sample appearance of color change is not large, to reach 400 ℃ when the sample shows light yellow, 520 ℃ sample yellow deterioration, the edge of the gray-white color. The damaged state for steel fiber concrete (Examples with steel fiber content *V*_*f*_ = 0.5 per cent and *V*_*f*_ = 1.5 per cent) under high temperature conditions is shown in Fig. 2.

## Principle of IWOA algorithm and simulation experiments

### Whale optimisation algorithm WOA

**Fig. 3:**
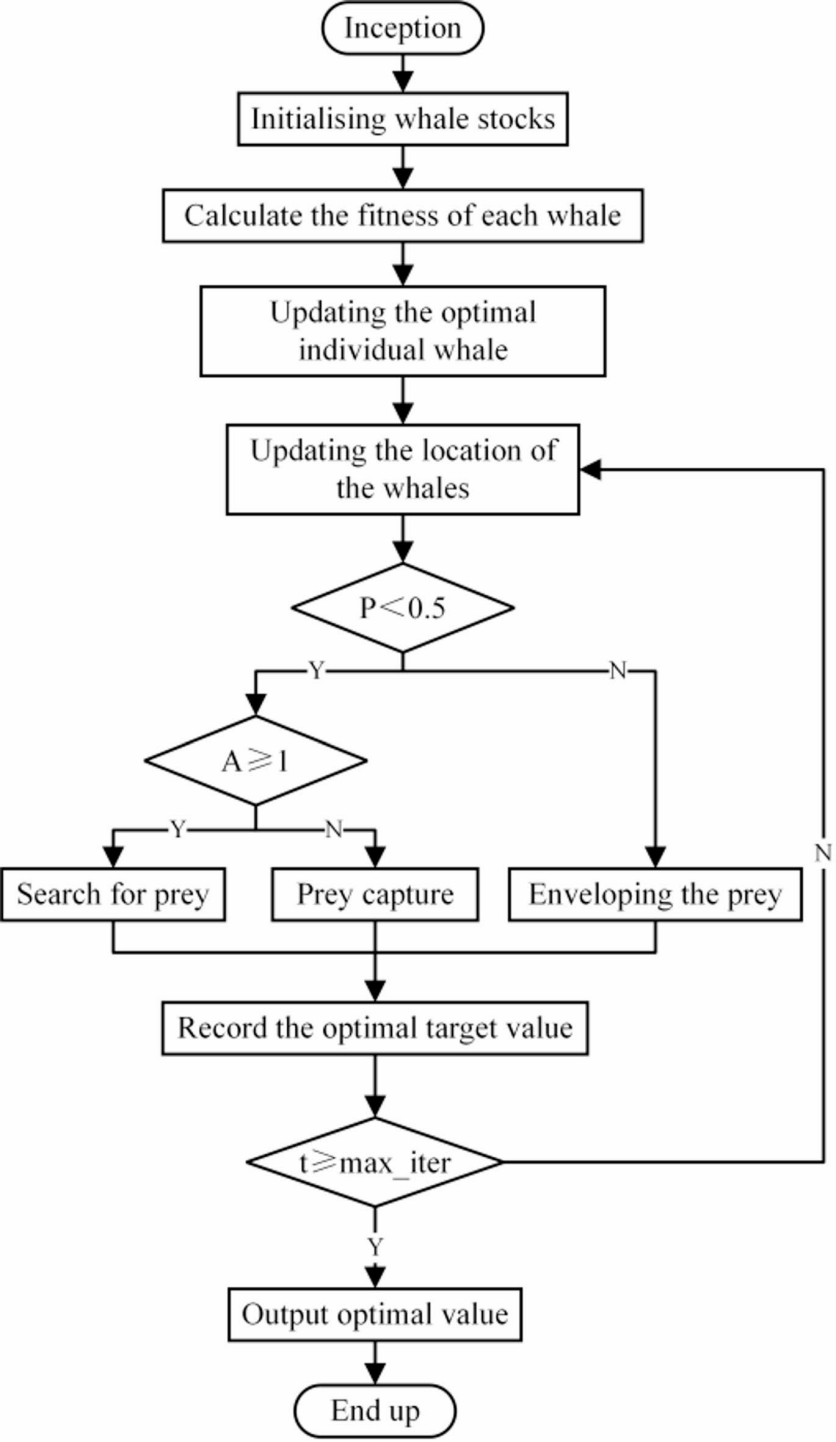
Flowchart of the WOA algorithm.

Whale Optimisation Algorithm WOA is a meta-heuristic optimisation algorithm developed by Mirjalilii et al.^35^. The algorithm imitates the 3 main feeding behaviours of whales: encircling prey, hunting and foraging. Whales will first search and gradually gain relevant information about their preys, then encircle their preys and update their information in a spiral until they find their preys, in other words, find an optimal way to solve the problem. The flowchart of the WOA algorithm is shown in Fig. 3.

#### Encircling prey

Whales can communicate with each other to learn about and surround their prey while they’re feeding. In the search space, the global optimal position is unknown, so the position of the individual whale closest to the prey is assumed to be the approximate optimal solution, with the other whales attempting to update their positions by approaching the enclosure to the optimal whale’s position. This encircling strategy allows the algorithm to search in a smaller spatial area, improving the algorithm’s search efficiency. The mathematical model for the encircling search is:

1 $$\:\begin{array}{c}D=\left|C{\cdot\:X}^{*}\left(t\right)-X\left(t\right)\right|\end{array}$$

2 $$\:\begin{array}{c}X\left(t+1\right)={X}^{*}\left(t\right)-A\cdot\:D\end{array}$$

where *t* represents the iteration number, $\:{X}^{*}\left(t\right)$ represents the position vector for the best individual whale to date, and $\:X\left(t\right)$ represents the position vector for the remaining whales. *A* and *C* are coefficient vectors and the position of $\:X\left(t\right)$ around the optimal solution is found by adjusting the values of *A* and *C*. When |*A*|≥1, the algorithm widens the scope and performs a global search. When |*A*|<1, the algorithm restricts the scope of the search and performs a local search. Formulas for coefficient vectors *A* and *C* are as follows:

3 $$\:\begin{array}{c}A=2a\cdot\:r-a\end{array}$$

4 $$\:\begin{array}{c}C=2\cdot\:r\end{array}$$

where: $\:r$∈[0,1], and *a* is a controlling parameter with *a* linearly decreasing in value from 2 to 0 as the whale population iterates through predation.

#### Bubble network attack

During feeding, whales hover and rise with their prey at the centre, spitting out bubbles to form an encirclement moving towards the prey. In WOA, the shrink mechanism and spiral update position mechanism were designed to describe bubble net hunting behaviour. This pattern of behaviour helps to prevent the algorithm from getting stuck in local optima and allows it to search for optimal solutions more quickly. Reducing the wraparound mechanism is accomplished through decreasing the value of *a* in Eq. (3), and the mathematical model for spiral updating of individual positions is as follows:

5 $$\:\begin{array}{c}X\left(t+1\right)=\left\{\begin{array}{c}{\:X}^{*}\left(t\right)-A\cdot\:D\:\:\:\:\:\:\:\:\:\:\:\:\:\:\:\:\:\:\:\:\:\:\:\:\:\:p<0.5\\\:\:{X}^{*}\left(t\right)+D\cdot\:{e}^{bl}\cdot\:{cos}\left(2\pi\:l\right)\:\:\:p\ge\:0.5\end{array}\right.\end{array}$$

where: $\:l$ represents a random number from [−1,1], with *p* used to distinguish which method to use for the position updating, *p*∈[0,1]. *b* represents a constant factor used to determine the shape of the logarithmic spiral.

#### Search for prey

The algorithm implemented the global search strategy to simulate the behaviour of a whale in search of prey, jumping out of local optimal solutions and exploring a wider solution space. Taking the whale at position $\:{X}^{{\prime\:}}\left(t\right)$ as an example, the equation of motion for searching for prey is:

6 $$\:\begin{array}{c}X\left(t+1\right)={X}^{{\prime\:}}\left(t\right)-A\cdot\:D\end{array}$$

7 $$\:\begin{array}{c}D=\left|C\cdot\:{X}^{{\prime\:}}\left(t\right)-X\left(t\right)\right|\end{array}$$

where $\:{X}^{{\prime\:}}\left(t\right)$ denotes a random location vector of whales.

### Improvement of Whale search algorithm

**Fig. 4:**
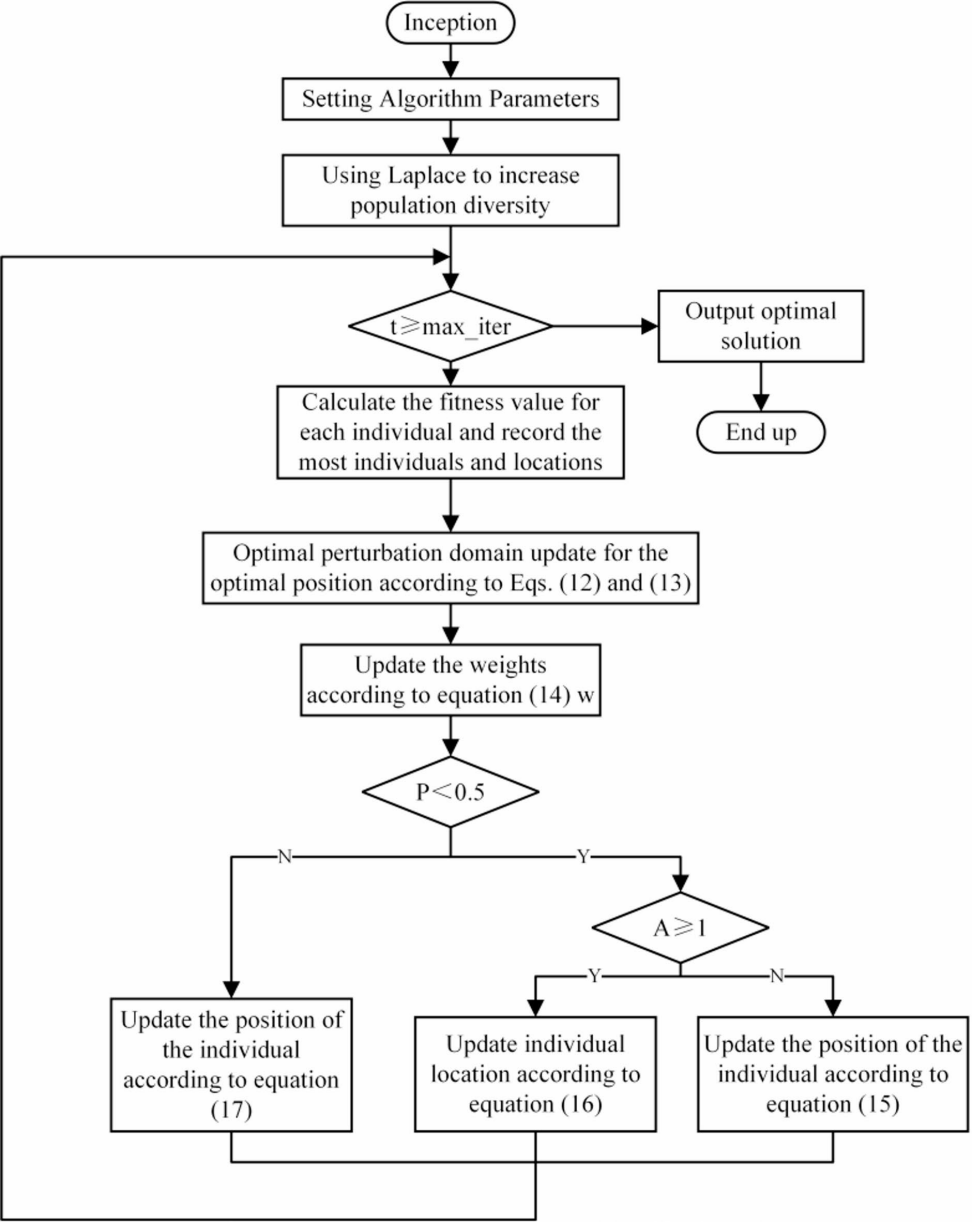
Flowchart of IWOA.

The tendency of the WOA algorithm to slip into partial optima and the simplicity of the parameter tuning methods within the model make it difficult to balance the ability to explore globally with the ability to exploit locally. Therefore, this paper presents an improved WOA algorithm, with the following specific improvement steps: (1) The Laplace crossover operator is introduced to increase the population diversity, solve the slow convergence and improve the algorithm’s optimisation accuracy. (2) Introducing the optimal neighbourhood perturbation strategy to improve the algorithm’s capacity to escape from the local optimum and avoid the phenomenon of premature maturity. (3) Use the adaptive weighting strategy to gradually strengthen the influence on the optimal location and enhance the convergence speed algorithm to some extent. (4) Introduce the notion of variable spiral search to expand the whale’s capacity to explore unexplored regions, hence improving the algorithm’s global search capabilities. Effective improvements are proposed from four aspects, namely, Laplace cross operator strategy, optimal neighbourhood perturbation strategy, adaptive weight strategy, and variable spiral position update strategy, to improve performance and efficiency for the WOA algorithm. The flowchart of the IWOA algorithm is shown in Fig. 4.

#### Laplace cross operator strategy

The Laplace crossover operator, proposed by Deep et al.^36^, improves the ability of global search and removes the local extremum constraints by performing dynamic crossover operations on the optimal whale positions obtained in each iteration, and generating children farther away from the parent in the pre-iteration period. Late iterations produce children closer to the parent, refining the search range and improving the solution accuracy. The Laplace density function and crossover calculation formulae are as follows:

8 $$\:\begin{array}{c}f\left(x\right)=\frac{1}{2b}{e}^{-\frac{\left|x-a\right|}{b}},-\infty\:<x<+\infty\:\end{array}$$

9 $$\:\begin{array}{c}{U}_{1i}={x}_{1i}+\beta\:\left|{x}_{1i}-{x}_{2i}\right|\end{array}$$

10 $$\:\begin{array}{c}{U}_{2i}={x}_{2i}+\beta\:\left|{x}_{1i}-{x}_{2i}\right|\end{array}$$

Where: $\:{U}_{1i}$ and $\:{U}_{2i}$ are the individual positions of the progeny generated by the crossover of the Laplace operator; *a*∈*R* represents the position parameters; *b* represents the scale parameters; $\:{x}_{1i}$ and $\:{x}_{2i}$ are the positions of the two individuals with the highest fitness in the solution space, respectively; and $\:\beta\:$ is the distribution random number. The improved whale optimisation algorithm position update formula is:

11 $$\:\begin{array}{c}X\left(t+1\right)=\left\{\begin{array}{c}{X}^{*}\left(t\right)+{(X}^{*}\left(t\right)-X\left(t\right))\cdot\:exp(-abs\left({r}_{1}\right))+(1-{r}_{2})\cdot\:(\stackrel{-}{X}\left(t\right)-X\left(t\right)),\:t<\frac{{t}_{max}}{2}\\\:{X}^{*}\left(t\right)+{(X}^{*}\left(t\right)-X\left(t\right))\cdot\:\frac{{exp}\left(-abs\left({r}_{1}\right)\right)}{2}+(1-{r}_{2})\cdot\:(\stackrel{-}{X}\left(t\right)-X\left(t\right)),\:t\ge\:\frac{{t}_{max}}{2}\end{array}\right.\end{array}$$

where: $\:{t}_{max}$ represents the maximal number of iterations, $\:{r}_{1}$, $\:{r}_{2}$∈[0,1] are random numbers uniformly distributed, and $\:\stackrel{-}{X}\left(t\right)$ is the average of all positions.

#### Optimal neighbourhood perturbation strategy

Since in the iterative process of WOA optimization algorithm, the optimal position is updated only when the optimal limit is exceeded, however, the reduction in the number of updates will result in a decrease in prediction accuracy of the algorithm, the optimal neighborhood perturbation strategy is implemented to prioritize the random searching near the optimal whale position, and then search for the optimal global value, which helps to jump out of the local optimum.

The neighbourhood perturbation formula is shown below, where $\:\stackrel{\sim}{X}\left(t\right)$ represents the updated position, which is updated to the global optimum if the produced position is superior to the original position, and vice versa if it remains unchanged.

12 $$\:\begin{array}{c}\stackrel{\sim}{X}\left(t\right)=\left\{\begin{array}{c}{X}^{*}\left(t\right)+0.5\cdot\:randn\cdot\:{X}^{*}\left(t\right),{p}_{1}<0.5\\\:{\:X}^{*}\left(t\right),{p}_{1}\ge\:0.5\:\:\:\:\:\:\:\:\:\:\:\:\:\:\:\:\:\:\:\:\:\:\:\:\:\:\:\:\:\:\:\:\:\:\:\:\:\:\:\:\:\end{array}\right.\end{array}$$

where: $\:randn$ is the generated random number obeying normal distribution, $\:{p}_{1}$ is a uniformly random number between [0,1]; $\:\stackrel{\sim}{X}\left(t\right)$ is the generated new position.

A greedy strategy is applied to the generated neighbourhood locations to decide whether to retain them or not, using the following formula:

13 $$\:\begin{array}{c}{X}^{*}\left(t\right)=\left\{\begin{array}{c}\stackrel{\sim}{X}\left(t\right),f\left(\stackrel{\sim}{X}\left(t\right)\right)\le\:f\left({X}^{*}\left(t\right)\right)\\\:{X}^{*}\left(t\right),f\left({X}^{*}\left(t\right)\right)\le\:f\left(\stackrel{\sim}{X}\left(t\right)\right)\end{array}\right.\end{array}$$

Where: $\:f\left(x\right)$ is the positional adaptation value of $\:x$.

#### Adaptive weighting strategies

To ensure the capability of the whale optimisation algorithm to seek both global and local optimisations, adaptive weights *w*, which vary with the increasing iteration number, are added to the location update in order to gradually increase the influence of the optimal location in the algorithm. If the weights are larger, the algorithm has a stronger global optimisation capability, which is conducive to global search; if the weights are smaller, it has a stronger local optimisation capability, which can converge quickly and guarantee the accuracy of the results. Based on the change of numbers of updates in the whale optimisation algorithm, the weights of the adaptive inertia, consisting of the numbers of iterations *t*, are selected as following:

14 $$\:\begin{array}{c}w=\left\{\begin{array}{c}0.2{cos}\left(\frac{\pi\:}{2}\cdot\:\left(1-\frac{t}{{t}_{max}}\right)\right)\:\:{p}_{2}>0.5\\\:0.2{sin}\left(\frac{\pi\:}{2}\cdot\:\left(1-\frac{t}{{t}_{max}}\right)\right)\:\:{p}_{2}\le\:0.5\end{array}\right.\end{array}$$

Dynamic adjustment of *w* size allows individual whales to be closely associated with the optimal whale position, even at various moments, while ensuring that other populations of whales can converge to the optimal position as quickly as possible. The improved whale optimisation algorithm position update formula is:

15 $$\:\begin{array}{c}X\left(t+1\right)=\left\{\:\begin{array}{c}w\cdot\:{X}^{*}\left(t\right)-A\cdot\:\left|C\cdot\:{X}^{*}\left(t\right)-X\left(t\right)\right|,\:p<0.5\\\:w\cdot\:{X}^{*}\left(t\right)+D\cdot\:{e}^{bl}{cos}\left(2\pi\:l\right),\:p\ge\:0.5\:\:\:\:\:\:\:\:\:\end{array}\right.\end{array}$$

16 $$\:\begin{array}{c}X\left(t+1\right)=w\cdot\:{X}^{{\prime\:}}\left(t\right)-A\cdot\:\left|C\cdot\:{X}^{{\prime\:}}\left(t\right)-X\left(t\right)\right|\end{array}$$

#### Update strategy for variable helix positions

When seeking for prey, whales adjust the moving distance for each position update dependent on the form of the spiral connecting the target’s location to their own. In Eq. (5), *b* is generally set as a constant, which leads to the whale’s spiral movement in searching for prey being too homogeneous, and very easily falls into the misunderstanding of local optimal solution. Therefore, this paper sets the parameter *b* as a parameter that varies with the numbers of iterations according to the idea of variable spiral search. After combining the adaptive weights, the new formula for updating the spiral position is as follows:

17 $$\:\begin{array}{c}\left\{\begin{array}{c}\:X\left(t+1\right)=w\left(t\right){X}^{*}\left(t\right)+bD\cdot\:{e}^{l}{cos}\left(2\pi\:l\right)\\\:\:b={e}^{5\cdot\:{cos}\left(\pi\:\cdot\:\left(1-\frac{t}{{t}_{max}}\right)\right)}\:\:\:\:\:\:\:\:\:\:\:\:\:\:\:\:\:\:\:\:\:\:\:\:\:\:\:\:\:\:\:\:\:\:\end{array}\right.\end{array}$$

### Simulation experiments

In this paper, to verify the optimisation search performance of the IWOA algorithm, we selected 15 benchmark test functions from the CEC2005. For the single-peak problem, *f*_1_-*f*_5_, these functions have a global minimum and no local minima, and are frequently employed to verify the convergence of an algorithm, as illustrated in Table 1. For the fundamental multi-peak problem, *f*_6_、*f*_7_、*f*_9_-*f*_11_, which are functions with multiple locally optimum solutions, are employed to predict the balanced capability of this algorithm to explore the decision space globally and exploit it locally, as seen in Table 2. For the expanded multi-peak and composite hybrid problems, *f*_13_、*f*_15_、*f*_21_-*f*_23_, which are more complex functions with multiple global and local optimal solutions, are employed to check the function performance when dealing with hybrid optimising problems, as shown in Tables 3 and 4.

**Table 1:** Functions for the single-peak problem.

Function	*D*	Search space	$\it \:{\text{f}}_{\text{m}\text{i}\text{n}}$
$\:{f}_{1}\left(x\right)={\sum\:}_{i=1}^{n}{x}_{i}^{2}$	30	$\:{\left[-\text{100,100}\right]}^{D}$	0
$\:{f}_{2}\left(x\right)={\sum\:}_{i=1}^{n}\left|{x}_{i}\right|+{\prod\:}_{i=1}^{n}\left|{x}_{i}\right|$	30	$\:{\left[-\text{10,10}\right]}^{D}$	0
$\:{f}_{3}\left(x\right)={\sum\:}_{i=1}^{n}({\sum\:}_{j-1}^{i}{x}_{j}{)}^{2}$	30	$\:{\left[-\text{100,100}\right]}^{D}$	0
$\:{f}_{4}\left(x\right)={{max}}_{i}\{\left|{x}_{i}\right|,1\le\:i\le\:n\}$	30	$\:{\left[-\text{100,100}\right]}^{D}$	0
$\:{f}_{5}=\sum\:_{i=1}^{n-1}\left[100{\left({x}_{i+1}-{x}_{i}^{2}\right)}^{2}+{\left({x}_{i}\right)-1}^{2}\right]$	30	$\:{\left[-\text{30,30}\right]}^{D}$	0

**Table 2:** Basic multi-peak problem functions.

Function	*D*	Search space	$\it \:{\text{f}}_{\text{m}\text{i}\text{n}}$
$\:{f}_{6}=\sum\:_{i=1}^{n}{\left([{x}_{i}+0.5]\right)}^{2}$	30	$\:{\left[-\text{100,100}\right]}^{D}$	0
$\:{f}_{7}\left(x\right)={\sum\:}_{i=1}^{n}i{{x}_{i}}^{4}+random\left[\text{0,1}\right)$	30	$\:{\left[-\text{1.28,1.28}\right]}^{D}$	0
$\:{f}_{9}\left(x\right)={\sum\:}_{i=1}^{n}\left[{x}_{i}^{2}-10{cos}\left(2\pi\:{x}_{i}\right)+10\right]$	30	[−5.12,5.12]^*D*^	0
$\:{f}_{10}\left(x\right)=-20{exp}(-0.2\sqrt{\frac{1}{n}{\sum\:}_{i=1}^{n}{{x}_{i}}^{2}})-{exp}(\frac{1}{n}{\sum\:}_{i=1}^{n}{cos}(2\pi\:{x}_{i}\left)\right)+20+e$	30	$\:{\left[-\text{32,32}\right]}^{D}$	0
$\:{f}_{11}\left(x\right)=\frac{1}{4000}\sum\:_{i=1}^{n}{x}_{i}^{2}-\prod\:_{i=1}^{n}{cos}\left(\frac{{x}_{i}}{\sqrt{i}}\right)+1$	30	$\:{\left[-\text{600,600}\right]}^{D}$	0

**Table 3:** Extended multi-peak and hybrid composite problem functions.

Function	*D*	Search space	$\it \:{\text{f}}_{\text{m}\text{i}\text{n}}$
$\:{f}_{13}\left(x\right)={\sum\:}_{i=1}^{n}u\left({x}_{i},\text{5,100,4}\right)+$ $\:0.1\left\{{{sin}}^{2}(3\pi\:{x}_{1}\right)+{\sum\:}_{i=1}^{n-1}{\left({x}_{i}-1\right)}^{2}[1+{{sin}}^{2}(3\pi\:{x}_{i+1})]+({x}_{n}-1{)}^{2}[1+{{sin}}^{2}(2\pi\:{x}_{n})\left]\right\}$	30	$\:{\left[-\text{50,50}\right]}^{D}$	0
$\:{f}_{15}\left(x\right)={{\sum\:}_{i=1}^{11}\left[{a}_{i}-\frac{{x}_{i}\left({{b}_{i}}^{2}+{b}_{i}{x}_{2}\right)}{{{b}_{i}}^{2}+{b}_{i}{x}_{3}+{x}_{4}}\right]}^{2}$	4	$\:{\left[-\text{5,5}\right]}^{D}$	0.0003
$\:{f}_{p}\left(x\right)=-{\sum\:}_{i=1}^{q}{\left[\left(x-{a}_{i}\right){\left(x-{a}_{i}\right)}^{T}+{c}_{i}\right]}^{-1}$	4	[0,10]^*D*^	*O*

**Table 4:** Parameter combinations.

*p*	*q*	*O*
21	5	−10
22	7	−10
23	10	−10

### Analysis of test results

#### Algorithm iteration process

**Fig. 5:**
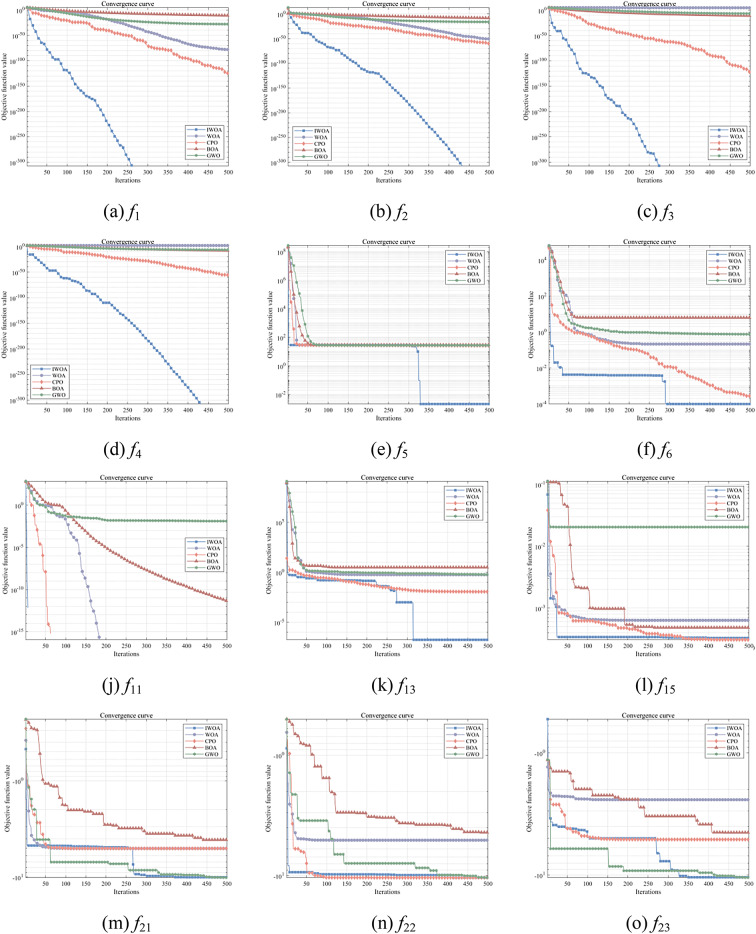
Convergence curve of the test function.

Crown Porcupine Optimization Algorithm (CPO) with unique perturbation strategy is competitive in complex multi-peaked problems. Butterfly Optimization Algorithm (BOA) is efficient in dealing with high dimensional nonlinear problems. Grey Wolf Optimization Algorithm (GWO) has fast convergence, simple parameters and stable performance in multi-domain optimization problems. The Whale Optimization Algorithm (WOA) is homologous to IWOA and widely used in engineering optimization problems. Therefore, in order to better analyse the performance of IWOA algorithm in terms of convergence speed, stability and optimization accuracy, WOA, CPO, BOA, GWO and IWOA are compared for test function optimization. Meanwhile, to ensure fairness in comparing each algorithm, the algorithms set the identical experimental parameters, the numbers of populations *N* = 30, the maximum numbers of iterations T = 500, as well as run 30 independent experiments for each algorithm. The results of the five algorithms are illustrated in Fig. 5.

From the convergence curves Fig. 5(a-e), it is obvious that in terms of convergence speed, the improved whale optimisation algorithm in this paper is faster than the WOA algorithm, CPO algorithm, BOA algorithm and GWO algorithm, and the IWOA algorithm completes the convergence between 250 and 450 iterations. In terms of convergence accuracy, the IWOA algorithm was able to achieve the optimum value of the test functions, while none of the other algorithms reached the optimum after 500 iterations. The IWOA algorithm has a better capability to find the optimum when dealing with single peak functions, and the iteration speed is fast and stable. According to Fig. 5(f-j), it is observed that the convergence rate of IWOA has an absolute advantage, and it can jump rapidly away from the local extreme value solution in the beginning of the iteration to reach the global optimum. For Fig. 5(h), IWOA reaches the global optimum after only 10 iterations. IWOA has faster convergence speed and better convergence accuracy when dealing with multi-peak functions. According to Fig. 5(k-o), it is obvious that WOA easily falls into the local optimum when coping with complex problems, while IWOA has a stronger global search ability, which also indicates that the improvement in this paper helps WOA leap out of the local optimum. For Fig. 5(l), IWOA achieves the algorithm’s global optimum in 25 iterations and CPO achieves the algorithm’s optimum in 370 iterations. This shows that IWOA has good coping ability in dealing with complex problem functions.

#### Algorithm evaluation indicators

**Fig. 6:**
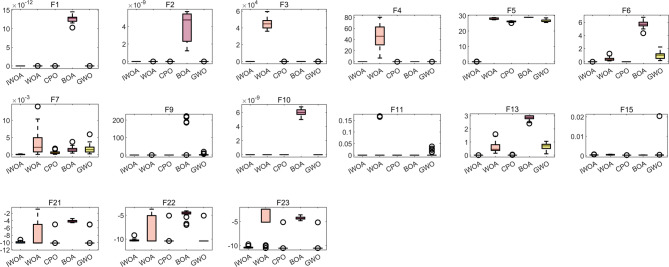
Box line diagram.

The assessment metrics for the intelligent optimisation algorithm’s data processing are the optimum, the standard deviation, the mean value and the worst value, and in order to observe the stability of the IWOA more graphically, a box-and-line diagram is plotted as can be seen in Fig. 6. The results of the assessment metrics of the 5 algorithms tested by the benchmark function are presented in Table 5. For the one-peak problem, the IWOA algorithm finds the theoretical optimum of the function on the functions *f*_1_-*f*_4_, whereas all of the other algorithms do not find the optimisation. The IWOA algorithm does not find the theoretical optimum for function *f*_5_, but the IWOA algorithm searches for the closest optimum compared to the other compared algorithms. For the multi-peak problem, the IWOA algorithm, the WOA algorithm, the CPO algorithm, and the BOA algorithm all obtain the theoretically optimal value of the function *f*_9_, whereas the standard, average and worst values of the IWOA algorithm and the CPO algorithm are all 0. The IWOA, WOA, CPO and GWO algorithms all find the theoretical optimum on function *f*_11_, whereas the standard, mean and worst values of the IWOA and CPO algorithms are 0. The IWOA algorithm searches for the optimum on functions *f*_6_, *f*_7_, and *f*_10_ more accurately than any of the other compared algorithms. In the expanded multi-peak problem and the hybrid compound problem, there is some error between the optimum found by the algorithm and the theoretical optimum, for example, for the function *f*_15_, the optimum found by the IWOA algorithm is 0.000317, whereas the theoretical optimum is 0.0003. On functions *f*_13_, *f*_21_ and *f*_22_, the IWOA algorithm searches for the optimal value with the lowest error between optimum and theoretical optimum comparing to the others comparative algorithms, thus having a high accuracy in finding the optimum value. The boxplots of the tests also show that the performance of the algorithm IWOA is relatively stable, with basically no outliers, and the medians are all close to the target optimum with very small deviations. In summary, the comparison of experimental results shows that IWOA has good convergence efficiency and robustness, and the algorithm performance is stable.

**Table 5:** Algorithm evaluation metrics.

		IWOA	WOA	CPO	BOA	GWO
*f*_*1*_	Best	0	3.19E-87	3.49E-145	1.03E-11	2.99E-29
	Std	0	6.52E-72	9.71E-101	8.74E-13	1.39E-27
	Avg	0	1.38E-72	1.77E-101	1.27E-11	8.94E-28
	Worse	0	3.54E-71	5.32E-100	1.45E-11	6.30E-27
*f*_*2*_	Best	0	5.80E-59	1.06E-72	1.22E-09	2.10E-17
	Std	0	4.77E-50	2.89E-54	1.54E-09	7.42E-17
	Avg	0	1.19E-50	5.65E-55	4.02E-09	1.06E-16
	Worse	0	2.53E-49	1.58E-53	5.71E-09	3.25E-16
*f*_*3*_	Best	0	3.60E + 04	6.50E-144	1.11E-11	1.45E-08
	Std	0	5.29E + 03	1.62E-95	7.51E-13	1.01E-04
	Avg	0	4.49E + 04	2.95E-96	1.26E-11	3.37E-05
	Worse	0	5.93E + 04	8.85E-95	1.40E-11	5.36E-04
*f*_*4*_	Best	0	6.6814	4.08E-72	5.31E-09	1.66E-08
	Std	0	20.4054	5.15E-51	3.58E-10	1.05E-06
	Avg	0	46.6667	1.70E-51	6.00E-09	8.44E-07
	Worse	0	79.9842	2.60E-50	6.71E-09	5.72E-06
*f*_*5*_	Best	1.17E-04	27.305	25.1652	28.893	25.5337
	Std	0.0361	0.4902	0.349	0.0161	0.8543
	Avg	0.0153	27.9996	26.1869	28.9241	26.9625
	Worse	0.1774	28.7567	26.6334	28.9522	28.57
*f*_*6*_	Best	1.27E-06	0.1311	4.83E-05	4.3353	0.177
	Std	2.31E-04	0.2604	1.19E-04	0.4872	0.4181
	Avg	9.69E-05	0.4286	1.63E-04	5.6894	0.903
	Worse	0.0013	1.2283	4.75E-04	6.7566	2.2428
*f*_*7*_	Best	2.24E-06	8.35E-05	1.05E-04	4.63E-04	2.45E-04
	Std	7.41E-05	0.0034	3.83E-04	6.91E-04	0.0012
	Avg	7.94E-05	0.0033	6.06E-04	0.0015	0.0017
	Worse	2.44E-04	0.014	0.0018	0.0037	0.0059
*f*_*9*_	Best	0	0	0	0	5.68E-14
	Std	0	4.15E-14	0	70.6614	4.2391
	Avg	0	7.58E-15	0	27.1477	2.2156
	Worse	0	2.27E-13	0	222.7362	19.1045
*f*_*10*_	Best	4.44E-16	4.44E-16	4.44E-16	4.98E-09	7.51E-14
	Std	0	2.22E-15	0	4.53E-10	1.82E-14
	Avg	4.44E-16	3.17E-15	4.44E-16	5.97E-09	1.03E-13
	Worse	4.44E-16	7.55E-15	4.44E-16	6.78E-09	1.39E-13
*f*_*11*_	Best	0	0	0	1.74E-12	0
	Std	0	0.0423	0	3.42E-12	0.0096
	Avg	0	0.0111	0	7.06E-12	0.0038
	Worse	0	0.1684	0	1.53E-11	0.0376
*f*_*13*_	Best	8.37E-07	0.1443	1.26E-05	2.4081	0.101
	Std	1.40E-04	0.3594	0.0087	0.166	0.2582
	Avg	8.04E-05	0.5961	0.0031	2.8552	0.6723
	Worse	5.37E-04	1.5941	0.044	3	1.0526
*f*_*15*_	Best	3.17E-04	3.09E-04	3.07E-04	3.14E-04	3.09E-04
	Std	8.90E-05	1.59E-04	4.41E-06	2.93E-05	0.0037
	Avg	4.10E-04	5.25E-04	3.09E-04	3.52E-04	0.001
	Worse	7.32E-04	7.75E-04	3.28E-04	4.20E-04	0.0204
*f*_*21*_	Best	−10.1492	−10.1525	−10.1532	−4.5914	−10.1529
	Std	0.2385	2.8274	0.9308	0.289	1.9184
	Avg	−9.9326	−7.6229	−9.9833	−4.1628	−9.308
	Worse	−9.2366	−0.882	−5.0552	−3.4528	−5.0552
*f*_*22*_	Best	−10.4017	−10.402	−10.4029	−7.0402	−10.4027
	Std	0.2386	2.4727	1.3485	0.6603	0.9701
	Avg	−10.2424	−8.93	−10.0486	−4.6832	−10.2238
	Worse	−9.1947	−3.7242	−5.0877	−4.0911	−5.0876
*f*_*23*_	Best	−10.536	−10.5288	−10.5364	−4.8207	−10.5363
	Std	0.1655	2.8091	0.9873	0.3065	0.9792
	Avg	−10.3828	−5.348	−10.3561	−4.3398	−10.3561
	Worse	−9.7306	−2.4156	−5.1285	−3.6016	−5.1716

## Model principles and evaluation metrics

### LSTM neural network model

**Fig. 7:**
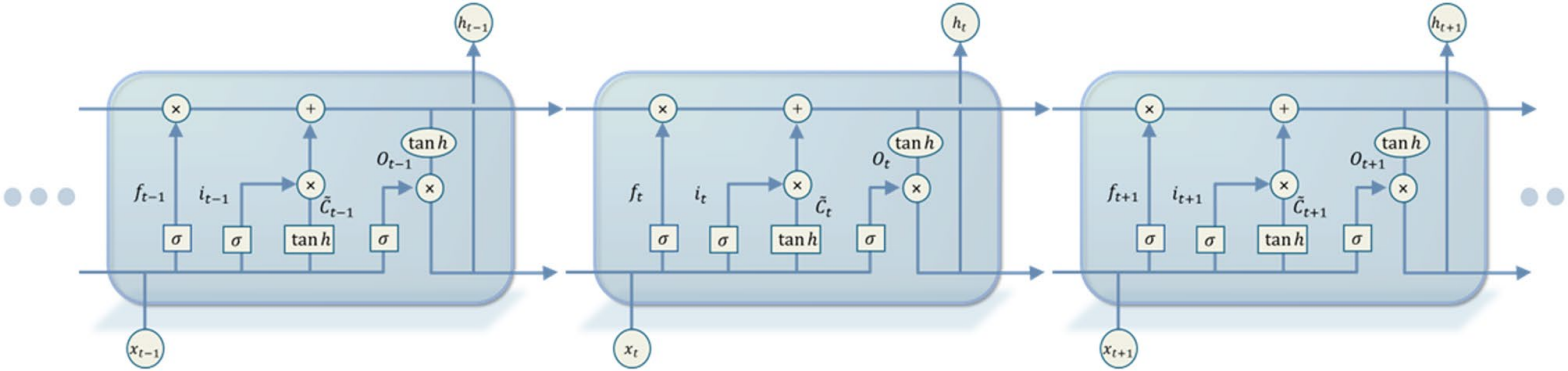
Structure of the LSTM model.

The LSTM is an optimisation of the recurrent neural network RNN, which creatively adds an input gate, a forget gate and an output gate to control the global memory metacells, primarily to solve the problems of gradient vanishing and gradient exploding when training long sequences. The structure of the LSTM model is illustrated in Fig. 7.

The forget gate filters the data information and uses the $\:\sigma\:$ function to discard information and retain information with a certain probability, and by adjusting the weights and biases of the forget gate, the training data fitting can be achieved. The input $\:{x}_{t}$ at the moment *t* and the output $\:{h}_{t-1}$ of the hidden layer at the moment *t-1* are output in the range of [0,1] through the $\:\sigma\:$ function, where 0 means all historical data information is completely discarded, and 1 means all historical data information is completely retained. The calculation formula is as follows:

18 $$\:\begin{array}{c}{f}_{t}=\sigma\:\left({W}_{f}\cdot\left[{h}_{t-1},{x}_{t}\right]+{b}_{f}\right)\end{array}$$

where *W*_*f*_ represents the forgetting gate weight matrix; *b*_*f*_ represents the forgetting gate bias term.

The input gate stores the transmitted information, processes the inputs of the current input sequence, and decides which information to keep to update the server’s state. The $\:{i}_{t}$ indicates the degree of trade-off for the newly added information, and the candidate unit $\:{c}_{t}$ is generated by the $\:{tan}h$ function, which is calculated as follows:

19 $$\:\begin{array}{c}{i}_{t}=\sigma\:\left({W}_{i}\cdot\left[{h}_{t-1},{x}_{t}\right]+{b}_{i}\right)\end{array}$$

20 $$\:\begin{array}{c}\stackrel{\sim}{{c}_{t}}={tan}h\left({W}_{c}\cdot\:\left[{h}_{t-1},{x}_{t}\right]+{b}_{c}\right)\end{array}$$

21 $$\:\begin{array}{c}{c}_{t}={f}_{t}{\cdot\:c}_{t-1}+{i}_{t}\cdot\:\stackrel{\sim}{{c}_{t}}\end{array}$$

where $\:{W}_{i}$ represents the weight matrix of the input gate and $\:{b}_{i}$ represents the corresponding bias term; $\:{W}_{c}$ represents the weight matrix of the neuron state and $\:{b}_{c}$ represents the corresponding bias term; $\:\stackrel{\sim}{{c}_{t}}$ is the variable for acquiring the new information, $\:{c}_{t}$ is the updated memory cell state variable, $\:{c}_{t-1}$ is the previous memory cell state variable, and $\:\sigma\:$ is the sigmoid activation function.

The output gate determines how much data information is output based on the original state of the new unit. *o*_*t*_ represents the degree of trade-off between the currently fused historical information and the input information, and *h*_*t*_ represents the data predicted by the output. This is calculated using the following formula:

22 $$\:\begin{array}{c}{o}_{t}=\sigma\:\left({W}_{o}\cdot\:\left[{h}_{t-1},{x}_{t}\right]+{b}_{o}\right)\end{array}$$

23 $$\:\begin{array}{c}{h}_{t}={o}_{t}\cdot\:{tan}h\left({c}_{t}\right)\end{array}$$

where $\:{W}_{o}$ represents the oblivious gate weight matrix; $\:{b}_{o}$ represents the oblivious gate bias term.

### Modelling IWOA-LSTM

**Fig. 8:**
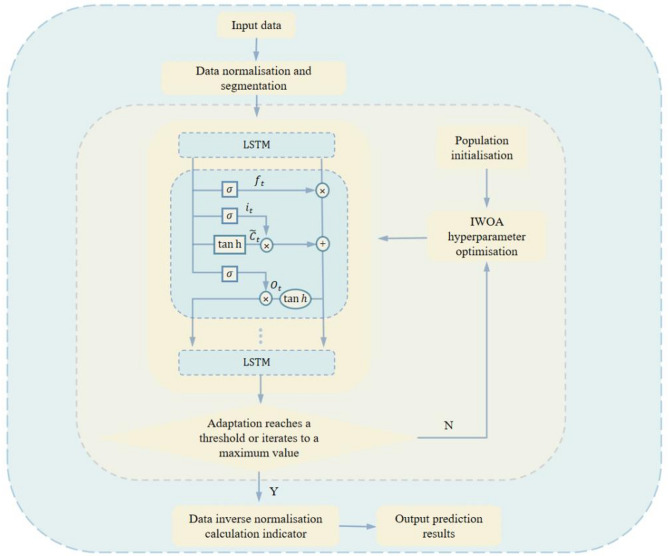
Flowchart of the IWOA-LSTM model.

An IWOA-LSTM model is developed to identify the constitutive model of fiber reinforced concrete at high temperatures. We use the IWOA algorithm to optimise the learning rate parameter, the number of hidden layer units and the regularisation coefficients in the LSTM units. The flowchart of the IWOA-LSTM model is shown in Fig. 8.

### Performance indicators

Three measures have been proposed by machine learning to assess a model’s prediction accuracy: coefficient of determination (*R*^*2*^), mean square error (*MSE*), and root mean square error (*RMSE*). These metrics are calculated by comparing the output values to the anticipated values. The following metrics are usually selected for evaluation.

(1)Formula for *MSE*:

24 $$\:\begin{array}{c}MSE=\frac{1}{n}\sum\:_{i=1}^{n}{\left({y}_{i}-{\stackrel{\sim}{y}}_{i}\right)}^{2}\end{array}$$

(2)Formula for *RMSE*:

25 $$\:\begin{array}{c}RMSE=\sqrt{\frac{\sum\:_{i=1}^{n}{\left({y}_{i}-{\stackrel{\sim}{y}}_{i}\right)}^{2}}{n}}\end{array}$$

(3)Formula for *R*^*2*^:

26 $$\:\begin{array}{c}{R}^{2}=1-\frac{\sum\:_{i=1}^{n}{\left({y}_{i}-{\stackrel{\sim}{y}}_{i}\right)}^{2}}{\sum\:_{i=1}^{n}{\left({y}_{i}-{\stackrel{-}{y}}_{i}\right)}^{2}}\end{array}$$

where $\:n$ represents the sample number, $\:{\stackrel{\sim}{y}}_{i}$ represents the fitted value, $\:{y}_{i}$ represents the true value, and $\:{\stackrel{-}{y}}_{i}$ represents the mean of the true value.

## Analysis of results

The destruction of concrete materials is the result of the expansion of microcracks within them, and the steady expansion and penetration of these ranks ultimately leads to the macroscopic destruction of concrete. The static damage of concrete is a time course and not an instantaneous event. Define the continuous damage $\:D$ as follows:

27 $$\:\begin{array}{c}D=\frac{{\sigma}_{0}-\sigma\:}{{\sigma}_{0}}=1-\frac{\sigma}{{\sigma}_{0}}\:\:(0\le\:D\le\:1)\end{array}$$

where $\:{\sigma\:}_{0}$ represents the stress of the perfect material without damage and $\:\sigma\:$ represents the stress of the real material containing damage.

In principle, the material damage develops with the deformation procedure, such that the damage *D* is a dependence of the strain $\:\epsilon\:$. Due to the loading environment, the evolution of material damage during loading is dependent on both the strain and the temperature, i.e. *D* = *D*($\:\epsilon\:$, *T*). The value of damage *D* can not be measured in the experiment directly, however, it should be considered that it can be measured by looking at the time *t* as an inverse function of *D*. Therefore, consider the inverse function of *t* with regard to *D* as damage. Constitutive relations, or the system recognition issue, are defined from the standpoint of systems science as determining the relationship between a system’s cause (input) and consequence (output). Therefore, the following formulation may be used to represent the one-dimensional constitutive relationships of steel fiber concrete at different temperatures:

28 $$\:\begin{array}{c}\sigma\:=f\left[\epsilon\:,T\right]\:\:\:\:\:\:\:\epsilon\:\le\:{\epsilon\:}_{th}\end{array}$$

29 $$\:\begin{array}{c}\sigma\:=f\left[\epsilon\:,T,D\right]=f\left[\epsilon\:,T,{t}^{-1}\left(D\right)\right]\:\:\:\:\:\:\epsilon\:>{\epsilon\:}_{th}\end{array}$$

where $\:{\epsilon\:}_{th}$ is the threshold strain (according to the relevant literature^37,38^ and experimental data, in this paper $\:{\epsilon\:}_{th}$ is taken as 0.75 times the peak strain), which represents the ultimate elastic strain.

### Model identification results

The settings of the relevant parameters of the IWOA-LSTM model are shown in Table 6.

**Table 6:** Optimal hyperparameters of the model.

Parameters	Space	Value
NumOfUnits	[2,100]	57
InitialLearnRate	[1e-05, 1e-03]	8.58e-04
L2Regularization	[1e-08, 1e-03]	4.46e-06
Optimizer		Adam

### Model identification results

**Fig. 9:**
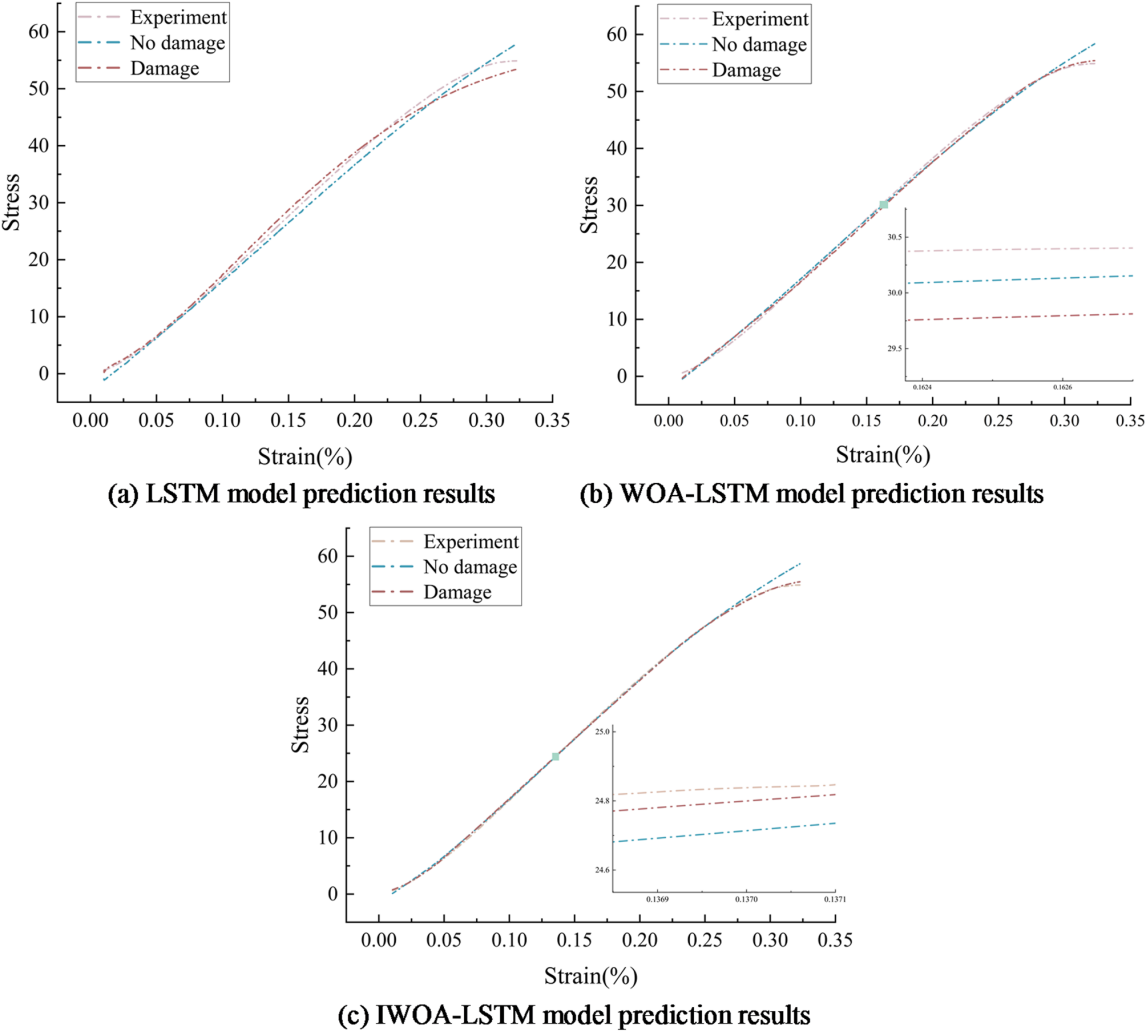
Comparison of model identification results.

Since test datasets constitute the foundation of all neural network models, the caliber of the gathered datasets greatly affects the model’s performance. In order to predict the stress-strain relationship of steel fiber concrete by the model and to avoid chance in the tests, 3–5 repetitions of the tests were carried out under each of the same conditions to ensure that at least three valid test results were obtained, with a standard deviation of the stress-strain curve of the specimens at each temperature of less than 5%, and that an average stress-strain curve was derived from the valid test results. Finally, the experimental curve closest to the average curve is selected for subsequent analysis to ensure a more reliable and consistent representation of the material behaviour. The data obtained from the uniaxial compression tests were put to use using neural networks in two cases. First case: strain $\:\epsilon\:$, temperature *T* as input and stress $\:\sigma\:$ as output (without considering damage evolution). Second case: strain $\:\epsilon\:$, temperature *T*, time *t* as inputs and stress $\:\sigma\:$ as output (considering damage evolution). The experimental data of steel fiber concrete (*V*_*f*_ =0.5%) is divided into training and test sets, 70% of the pre-processed data is used as training set and the remaining 30% is used as test set. A concrete specimen made of steel fibers at 400 °C as an illustration, test data were fed into the LSTM, WOA-LSTM, and IWOA-LSTM models, respectively. The models were trained to predict the steel fiber concrete stress-strain curves under high temperature conditions, and the steel fiber concrete stress-strain curves following high temperature were compared with the deep network model’s prediction. Figure 9 displays the results of the identification.

The LSTM model is the lowest constitutive identification model among these three, as seen in Fig. 9. The LSTM model’s projected curves do not closely match the actual curves when damage is taken into account; without damage, the predictions are lower than the current experimental curves. Comparing Fig. 9(a), it can be seen that the optimised LSTM model with the addition of WOA or IWOA has improved in verifying its accuracy in defining the damage, and both of them can define the macro-continuous damage, and the prediction curves match the experimental curves better after the addition of the damage data, from which the accuracy in defining the damage can be verified. In the range of $\:{\epsilon\:}_{th}$, the WOA-LSTM and IWOA-LSTM prediction curves match well with the test curves, whereas the concrete specimen deformation deviates from the test and prediction curves after exceeding the bounds of the model learning, and we believe that it is the appearance of the damage that leads to the deviation of the curves, as shown by the blue dotted lines in Fig. 9(b-c). It is evident that there is a stress difference between the outcomes that are anticipated with and without consideration of damage evolution. This difference represents the weakening impact that damage evolution causes as strain increases. The threshold strain $\:{\epsilon\:}_{th}$ marks the start of damage evolution and is an important material parameter for designing the strength of steel fiber concrete in terms of damage evolution and fracture damage. After accounting for the damage evolution, i.e., entering the time *t* as an inverse function of the damage *D* for input, the predicted curves of the WOA-LSTM and IWOA-LSTM models agree well with the experimental curves over the whole strain range, as shown by the red underlined lines in Fig. 9(b-c).

### Model comparison

#### Comparison between WOA-LSTM and IWOA-LSTM

**Fig. 10:**
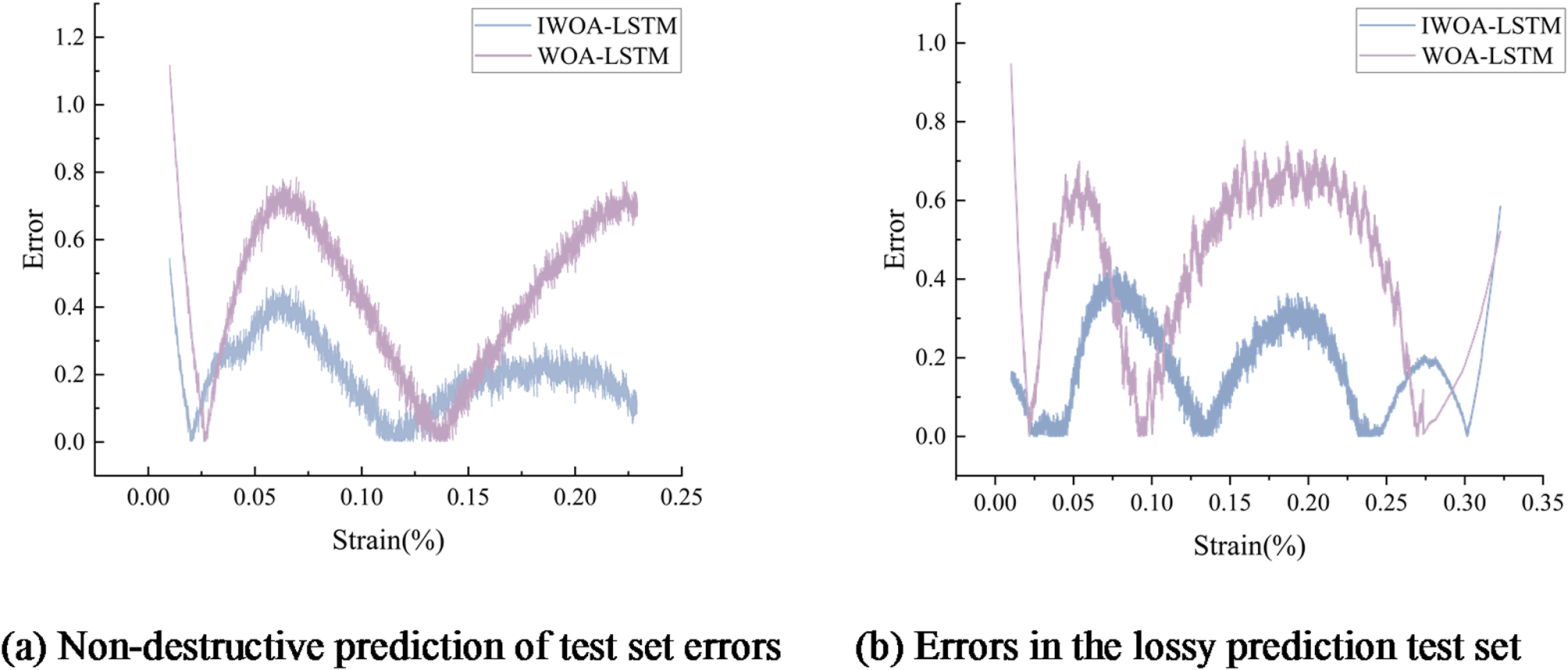
Results of error analysis.

Figure 9 shows that both algorithms, WOA-LSTM and IWOA-LSTM, can study the damage evolution law and predict the stress-strain curve of concrete materials. To compare the precision of the two algorithms throughout the prediction process, the prediction errors of the concrete materials were selected for comparison between the intrinsic identification process considering the damage evolution and without considering the damage evolution. Figure 10 presents the findings.

30 $$\:\begin{array}{c}E=\left|\stackrel{\sim}{\sigma\:}-\sigma\:\right|\end{array}$$

where $\:E$ represents the prediction error, $\:\stackrel{\sim}{\sigma\:}$ represents the predicted stress and $\:\sigma\:$ represents the experimental stress.

As shown in Fig. 10, the prediction error of IWOA-LSTM basically stays within 0.4, while the prediction error of WOA-LSTM is within 1.2 and is very unstable. In the SFRC constitutive identification with and without considering damage, the prediction errors of both the WOA-LSTM and IWOA-LSTM models are larger at both the beginning and end of loading, mainly due to the fact that only one side of the data exists at both the beginning and end of loading. In terms of prediction findings, both with considering and without damage evolution, the IWOA-LSTM model suggested in this study is very accurate in forecasting the stress-strain behaviour of concrete after high temperature with minimal error. Therefore, it can be verified that the damage defined by IWOA-LSTM is more reliable.

#### Comparative analysis of model indicators

**Fig. 11:**
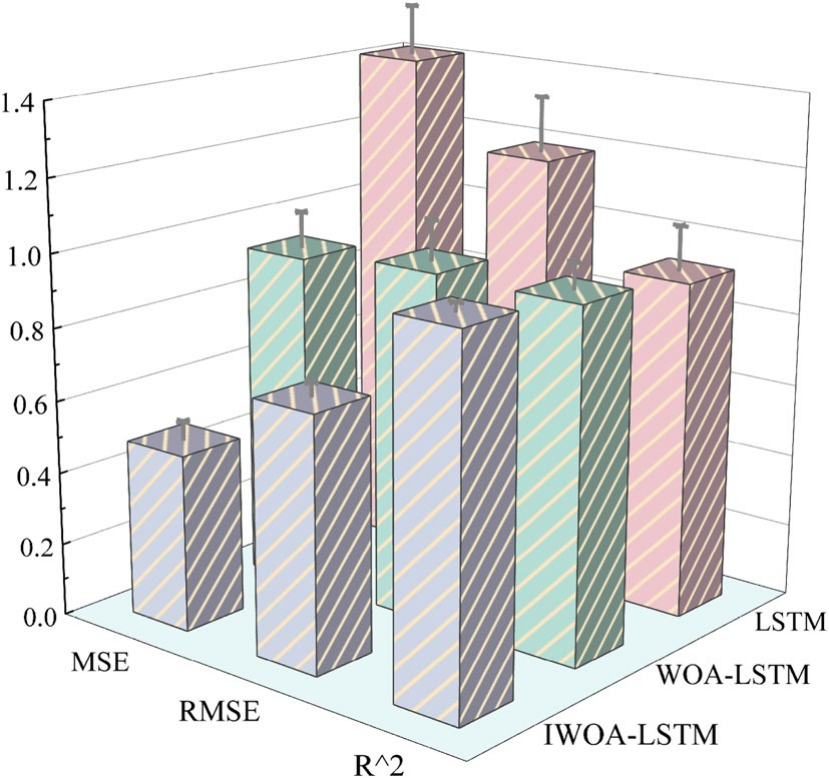
Comparison and visualisation of model indicators.

The prediction outcomes are compared to evaluate the effectiveness of the proposed LSTM, WOA-LSTM, and IWOA-LSTM models. Table 7 displays the average comparison results of ten runs of each model, whereas Fig. 11 displays the visualisation results.

**Table 7:** Comparison of indicators for Models.

	LSTM	WOA-LSTM	IWOA-LSTM
*MSE*	1.4151	0.9303	0.4868
*RMSE*	1.1896	0.9645	0.6977
*R* ^*2*^	0.9260	0.9624	0.9981

The metrics for errors *MSE* and *RMSE* are metrics used to evaluate a model’s prediction accuracy. The IWOA-LSTM model has the lowest error and the best prediction accuracy, whereas the LSTM model has the most error, as shown in Table 6; Fig. 11. For the most commonly used *MSE* assessment metrics, the IWOA-LSTM model is 47.66% more accurate than the WOA-LSTM model and 65.60% more accurate than the LSTM model. The degree of correlation between anticipated and true values, or *R*^*2*^, is a measure of the model’s goodness-of-fit. The closer the number is to 1, the better the model predicts the data. The IWOA-LSTM model has the *R*^*2*^ is the highest, reaching more than 0.99. In summary, the IWOA-LSTM model has better performance.

### Validation of the IWOA-LSTM

**Fig. 12:**
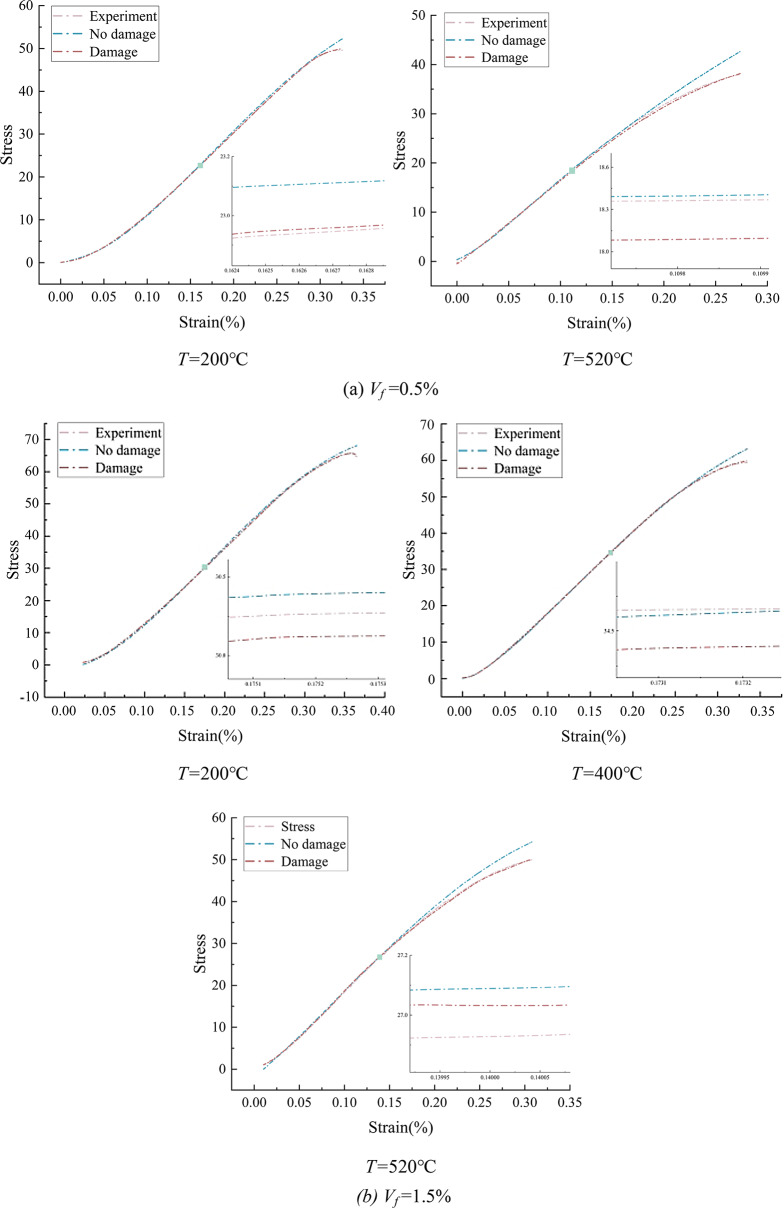
Recognition results of SFRC.

Instead of relying on conventional mathematical formulas and fundamental laws of mechanics, the IWOA-LSTM based constitutive behaviour of concrete relies on the inputs and outputs of different modes for constitutive identification. Steel fiber concrete specimens with *V*_*f*_ =0.5% (*T* = 200℃ and *T* = 520℃) and *V*_*f*_ =1.5% (*T* = 200℃, *T* = 400℃, and *T* = 520℃) were chosen in order to further confirm the IWOA-LSTM model’s reliability. Sample test data were then entered into the model, with 70% serving as the training set and 30% as the test set. In Fig. 12, the prediction results are displayed.

**Fig. 13:**
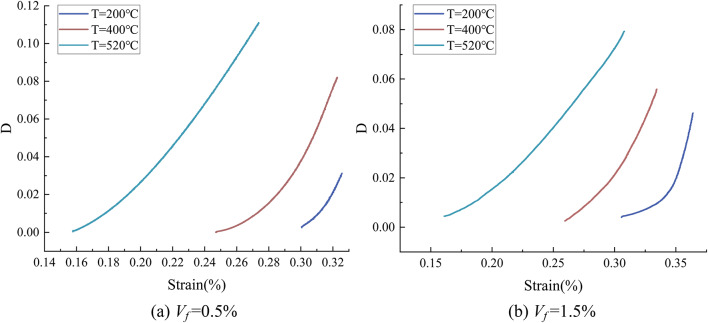
Damage evolution curve.

The prediction findings in Fig. 12 show that the IWOA-LSTM model’s prediction results are quite close to the experimental curves when damage is taken into consideration. The temperature affects the damage evolution process in addition to strain, and both temperature and strain determine continuous damage. The appearance of the evolution of the damage *D* with the strain at different temperatures is produced as shown in Fig. 13 by comparing and analyzing the experimental curves and the projected curves without taking the damage into consideration. As the loading process goes on, the damage variable varies. At low stress levels, there is no obvious damage; as external loads increase, the damage value climbs progressively; and as temperature rises, the threshold strain, or $\:{\epsilon\:}_{th}$, gets smaller and smaller.

## Conclusion

In this study, we present an Improved Whale Algorithm Optimised Long Short-Term Memory neural network-based concrete constitutive identification model (IWOA-LSTM identification model). The constitutive identification model of steel fiber concrete at elevated temperatures is used to determine the damage evolution law and constitutive response of steel fiber concrete by using the IWOA-LSTM model with different input-output modes based solely on the experimental data, without making any prior assumptions on their constitutive relationships. The following are the primary conclusions:

1. Targeting the fundamental whale optimization method, which is sluggish to converge and prone to local optimisation. To address these initial shortcomings, broaden the population’s diversity, and improve the balance between local exploitation and global search, four new strategies are introduced: the Laplace cross operator strategy, the optimal neighbourhood perturbation strategy, the adaptive weighting strategy, and the variable helix position update strategy. It extends the whale optimization algorithm’s global search capabilities, which improves the algorithm’s stability and quality of solution, in addition to increasing the algorithm’s convergence speed and accuracy of solutions.
2. The five algorithms are tested on 15 benchmark test functions. The improved WOA algorithm in this paper has stronger efficiency and accuracy, as evidenced by the convergence curve, which shows that the WOA algorithm is easy to fall into the local optimum while the IWOA algorithm converges quickly and can overcome the restriction of the local optimum solution to obtain higher solving accuracy. It can also be seen from the boxplots of the test that compared to the WOA algorithm, CPO algorithm, BOA algorithm and GWO algorithm, the IWOA algorithm has a more stable performance, basically no outliers, and the medians are close to the target optimal value, with very small deviations, which provides a strong stability.
3. In this paper, three constitutive identification models, LSTM, WOA-LSTM and IWOA-LSTM, are developed to decouple the rheology from the damage by identifying the constitutive model of steel-fiber concrete at high temperatures without making any assumptions. Based on the simulation results of the models on the test and training sets, it is clear that the IWOA-LSTM model outperforms the WOA-LSTM and LSTM models in terms of predicting the stress-strain relationship of concrete after exposure to high temperatures. The correlation coefficient, mean square error, and root mean square error of the IWOA-LSTM model are 0.9981, 0.4868, and 0.6977, respectively. The mean square error of the IWOA-LSTM model is 47.66% and 65.60% more accurate than that of the WOA-LSTM model and the LSTM model, respectively, and it has a higher computational accuracy.
4. The IWOA-LSTM neural network model was applied to identify steel fiber concrete at various temperatures (200℃, 400℃, and 520℃), resulting in damage evolution curves for SFRC. The findings indicate that no appreciable damage occurs at low stress levels, that damage values progressively rise with increasing external loads, and that $\:{\epsilon\:}_{th}$ falls with rising temperatures.
5. Based on machine learning to determine the ontological model of the material, does not rely on the failure mechanism that is not yet clear, does not need complex parameter analysis, through the given sample data to learn, directly extract the rules from it, to obtain the required data, in order to ensure the accuracy while saving a lot of time. The IWOA-LSTM model designed and developed in this paper has high accuracy and generalisation ability, which provides a new idea and option for future research. For future research, this paper only considers the high temperature principal structure identification of concrete under quasi-static conditions, and an attempt can be made to improve the model and carry out research on the high temperature principal structure identification of concrete under impact loading.

## Electronic supplementary material

Below is the link to the electronic supplementary material.

Supplementary Material 1

## Data Availability

Data supporting the results of this study are available from the corresponding author [S.D] and can be made available upon reasonable request.

## References

[1] Zhou, Z. et al. Inexpensive anti-icing concrete material for application to tunnel and slope engineering infrastructures in cold regions. *ACS Appl. Mater. Interfaces*. **13** (44), 53030–53045. 10.1021/acsami.1c14046 (2021). 34723465 10.1021/acsami.1c14046

[2] Zhang, W., Zheng, Q., Ashour, A. & Han, B. Self-healing cement concrete compo-sites for resilient infrastructures: A review. *Compos. Part. B: Eng.* **189**, 107892. 10.1016/j.compositesb.2020.107892 (2020).

[3] Huang, H., Gao, X., Li, L. & Wang, H. Improvement effect of steel fiber orientation control on mechanical performance of UHPC. *Constr. Build. Mater.* **188**, 709–721. 10.1016/j.conbuildmat.2018.08.146 (2018).

[4] Filkov, A. I. et al. A review of thermal exposure and fire spread mechanisms in large outdoor fires and the built environment. *Fire Saf. J.* 103871. 10.1016/j.firesaf.2023.103871 (2023).

[5] Zhang, X. et al. Experimental study on thermal hazard and facade flame characterization induced by incontrollable combustion of indoor energy usage. *Energy* **207**, 118173. 10.1016/j.energy.2020.118173 (2020).

[6] Hameed, R., Sellier, A., Turatsinze, A. & Duprat, F. Metallic fiber-reinforced concrete behaviour: experiments and constitutive law for finite element modeling. *Eng. Fract. Mech.* **103**, 124–131. 10.1016/j.engfracmech.2012.11.022 (2013).

[7] Wang, C., Yuan, J., Zhang, Y. & Ma, Z. Study on the mesoscopic mechanical behavior and damage constitutive model of micro-steel fiber reinforced recycled aggregate concrete. *Constr. Build. Mater.* **443**, 137767. 10.1016/j.conbuildmat.2024.137767 (2024).

[8] Yu, Y., Xu, J., Chen, W. & Wu, B. Mesoscale modeling of flexural fracture behav-eor in steel fiber reinforced concrete. *Adv. Struct. Eng.* **27** (4), 565–584. 10.1177/13694332241226921 (2024).

[9] Bi, Z. et al. Research on dynamic constitutive model of steel fiber reinforced concrete with different steel fiber content and matrix strength. *Constr. Build. Mater.* **433**, 136671. 10.1016/j.conbuildmat.2024.136671 (2024).

[10] Chen, C. et al. Dynamic constitutive modeling of steel fiber reinforced concrete considering damage evolution under high strain rate. *Constr. Build. Mater.* **440**, 137433. 10.1016/j.conbuildmat.2024.137433 (2024).

[11] Wen, Y. B., Huang, R. Y., Ma, J., Xiao, K. T. & ****, & Damage evolution equations for concrete materials under high temperature and high strain rate. *Chin. J. High. Press. Phys.* **35** (2). 10.11858/gywlxb.20200617 (2021).

[12] Nguyen, T., Kashani, A., Ngo, T. & Bordas, S. Deep neural network with high-order neuron for the prediction of foamed concrete strength. *Computer-Aided Civ. Infrastruct. Eng.* **34** (4), 316–332. 10.1111/mice.12422 (2019).

[13] Naderpour, H., Rafiean, A. H. & Fakharian, P. Compressive strength prediction of environmentally friendly concrete using artificial neural networks. *J. Building Eng.* **16**, 213–219. 10.1016/j.jobe.2018.01.007 (2018).

[14] Congro, M. et al. Prediction of the residual flexural strength of fiber reinforced concrete using artificial neural networks. *Constr. Build. Mater.* **303**, 124502. 10.1016/j.conbuildmat.2021.124502 (2021).

[15] Jiang, Y. et al. Predicti-on of time-dependent concrete mechanical properties based on advanced deep learning models considering complex variables. *Case Stud. Constr. Mater.* **21**, e03629. 10.1016/j.cscm.2024.e03629 (2024).

[16] Ta, Q. B., Huynh, T. C., Pham, Q. Q. & Kim, J. T. Corroded bolt identification using mask region-based deep learning trained on synthesized data. *Sensors* **22** (9), 3340. 10.3390/s22093340 (2022). 35591032 10.3390/s22093340 PMC9104359

[17] Kim, J. T., Ta, Q. B., Dang, N. L., Kim, Y. C. & Kam, H. D. Semantic crack-image identification framework for steel structures using atrous convolution-based Deeplabv3 + Network. *Smart Struct. Syst. Int. J.* **30** (1), 17–34. 10.12989/sss.2022.30.1.017 (2022).

[18] Fan, Y., Li, H., Bao, Y. & Xu, Y. Cycle-consistency-constrained few-shot learning framework for universal multi-type structural damage segmentation. *Struct. Health Monit.* 14759217241293467. 10.1177/14759217241293467 (2024).

[19] Ghaboussi, J., Garrett Jr, J. H. & Wu, X. Knowledge-based modeling of material behavior with neural networks. *J. Eng. Mech.* **117** (1), 132–153. 10.1061/(ASCE)0733-9399(1991)117:1(132) (1991).

[20] Zhang, N., Shen, S. L., Zhou, A. & Jin, Y. F. Application of LSTM approach for modelling stress–strain behaviour of soil. *Appl. Soft Comput.* **100**, 106959. 10.1016/j.asoc.2020.106959 (2021).

[21] Tanhadoust, A. et al. Predicting stress-strain behavior of normal weight and lightweight aggregate concrete exposed to high temperature using LSTM recurrent neural network. *Constr. Build. Mater.* **362**, 129703. 10.1016/j.conbuildmat.2022.129703 (2023).

[22] Li, P., Zhao, H., Gu, J. & Duan, S. Dynamic constitutive identification of concrete based on improved Dung beetle algorithm to optimize long short-term memory model. *Sci. Rep.* **14** (1), 6334. 10.1038/s41598-024-56960-z (2024). 38491105 10.1038/s41598-024-56960-z PMC11344044

[23] Wang, L. L., Hu, S. S., Yang, L., Dong, X. L. & Wang, H. Chatting about dynamic strength and damage evolution. *Explosion Shock*. **37** (2), 169–179. 10.11883/1001-1455(2017)02-0169-11 (2017).

[24] Xu, M. & Wang, L. A new method for studying the dynamic response and dama-ge evolution of polymers at high strain rates. *Mech. Mater.* **38** (1–2), 68–75. 10.1016/j.mechmat.2005.05.010 (2006).

[25] Wang, Z., Chen, Q., Wang, Z. & Xiong, J. The investigation into the failure criteria of concrete based on the BP neural network. *Eng. Fract. Mech.* **275**, 108835. 10.1016/j.engfracmech.2022.108835 (2022).

[26] Ning, J., Feng, Y., Ren, H. & Xu, X. Prediction model for the failure behavior of concrete under impact loading base on back propagation neural network. *Constr. Build. Mater.* **411**, 134297. 10.1016/j.conbuildmat.2023.134297 (2024).

[27] Zaidi, S. K. A., Ayaz, M. & Sharma, U. K. Unified model using artificial neural network for high strength fibrous concrete subjected to elevated temperature. *Innovative Infrastructure Solutions*. **7** (1), 87. 10.1007/s41062-021-00675-x (2022).

[28] Srivastava, S. & Lessmann, S. A comparative study of LSTM neural networks in forecasting day-ahead global horizontal irradiance with satellite data. *Sol. Energy*. **162**, 232–247. 10.1016/j.solener.2018.01.005 (2018).

[29] Xu, Z., Chen, J., Shen, J. & Xiang, M. Recursive long short-term memory network for predicting nonlinear structural seismic response. *Eng. Struct.* **250**, 113406. 10.1016/j.engstruct.2021.113406 (2022).

[30] Zhang, Y. C., Wang, Y. & Guo, K. Y. Prediction of gas outflow from working face based on WOA-LSTM. *Min. Saf. Environ. Prot.*, **50**(5), 50–55, 10.19835/j.issn.1008-4495.2023.05.008. (2023).

[31] Yan, X., Weihan, W. & Chang, M. Research on financial assets transaction predic-tion model based on LSTM neural network. Neural Computing and Applications, 33(1), 257–270, (2021). 10.1007/s00521-020-04992-7

[32] Jia, X. et al. Prediction of sea surface te-mperature in the East China sea based on LSTM neural network. *Remote Sens.* **14** (14), 3300. 10.3390/rs14143300 (2022).

[33] Venkatraj, V. & Dixit, M. K. Challenges in implementing data-driven approaches for Building life cycle energy assessment: A review. *Renew. Sustain. Energy Rev.* **160**, 112327. 10.1016/j.rser.2022.112327 (2022).

[34] Li, P., Liu, J., Duan, S. & Huang, R. Variation pattern of the compressive streng-th of concrete under combined heat and moisture conditions. *Materials* **16** (4), 1548. 10.3390/ma16041548 (2023). 36837182 10.3390/ma16041548 PMC9967774

[35] Mirjalili, S. & Lewis, A. The Whale optimization algorithm. *Adv. Eng. Softw.* **95**, 51–67. 10.1016/j.advengsoft.2016.01.008 (2016).

[36] Deep, K. & Thakur, M. A new crossover operator for real coded genetic algorithms. *Appl. Math. Comput.* **188** (1), 895–911. 10.1016/j.amc.2006.10.047 (2007).

[37] Bian, H., Liu, Y., Guo, Y., Liu, Y. & Shi, W. Investigating stress–strain relationship and damage constitutive model of basalt fiber reinforced concrete under uniaxial compression. *J. Building Eng.* **73**, 106789. 10.1016/j.jobe.2023.106789 (2023).

[38] Le, Q. X., Torero, J. L. & Dao, V. T. Stress–strain–temperature relationship for concrete. *Fire Saf. J.* **120**, 103126. 10.1016/j.firesaf.2020.103126 (2021).

